# GSK-3β Can Regulate the Sensitivity of MIA-PaCa-2 Pancreatic and MCF-7 Breast Cancer Cells to Chemotherapeutic Drugs, Targeted Therapeutics and Nutraceuticals

**DOI:** 10.3390/cells10040816

**Published:** 2021-04-06

**Authors:** Stephen L. Abrams, Shaw M. Akula, Akshaya K. Meher, Linda S. Steelman, Agnieszka Gizak, Przemysław Duda, Dariusz Rakus, Alberto M. Martelli, Stefano Ratti, Lucio Cocco, Giuseppe Montalto, Melchiorre Cervello, Peter Ruvolo, Massimo Libra, Luca Falzone, Saverio Candido, James A. McCubrey

**Affiliations:** 1 Department of Microbiology and Immunology, Brody School of Medicine at East Carolina University, Brody Building 5N98C, Greenville, NC 27858, USA; abramss@ecu.edu (S.L.A.); akulas@ecu.edu (S.M.A.); mehera19@ecu.edu (A.K.M.); lssteelman@gmail.com (L.S.S.); 2 Department of Molecular Physiology and Neurobiology, University of Wrocław, 50-335 Wrocław, Poland; agnieszka.gizak@uwr.edu.pl (A.G.); przemyslaw.duda@uwr.edu.pl (P.D.); dariusz.rakus@uwr.edu.pl (D.R.); 3 Department of Biomedical and Neuromotor Sciences, Università di Bologna, 40126 Bologna, Italy; alberto.martelli@unibo.it (A.M.M.); stefano.ratti@unibo.it (S.R.); lucio.cocco@unibo.it (L.C.); 4 Department of Health Promotion, Maternal and Child Care, Internal Medicine and Medical Specialties, University of Palermo, 90133 Palermo, Italy; giuseppe.montalto@unipa.it; 5 Institute for Biomedical Research and Innovation, National Research Council (CNR), 90133 Palermo, Italy; melchiorre.cervello@irib.cnr.it; 6 Department of Leukemia, MD Anderson Cancer Center, The University of Texas, Houston, TX 77030, USA; pruvolo@mdanderson.org; 7 Research Center for Prevention, Diagnosis and Treatment of Cancer (PreDiCT), University of Catania, 95123 Catania, Italy; mlibra@unict.it (M.L.); lucafk92@hotmail.it (L.F.); saverio1979@hotmail.it (S.C.); 8 Department of Biomedical and Biotechnological Sciences, University of Catania, 95123 Catania, Italy

**Keywords:** GSK-3β, targeted therapy, PDAC, breast cancer, KRas, β-catenin, BCL2, chemotherapeutic drugs, nutraceuticals

## Abstract

Glycogen synthase kinase-3 (GSK-3) is a regulator of signaling pathways. KRas is frequently mutated in pancreatic cancers. The growth of certain pancreatic cancers is KRas-dependent and can be suppressed by GSK-3 inhibitors, documenting a link between KRas and GSK-3. To further elucidate the roles of GSK-3β in drug-resistance, we transfected KRas-dependent MIA-PaCa-2 pancreatic cells with wild-type (WT) and kinase-dead (KD) forms of GSK-3β. Transfection of MIA-PaCa-2 cells with WT-GSK-3β increased their resistance to various chemotherapeutic drugs and certain small molecule inhibitors. Transfection of cells with KD-GSK-3β often increased therapeutic sensitivity. An exception was observed with cells transfected with WT-GSK-3β and sensitivity to the BCL2/BCLXL ABT737 inhibitor. WT-GSK-3β reduced glycolytic capacity of the cells but did not affect the basal glycolysis and mitochondrial respiration. KD-GSK-3β decreased both basal glycolysis and glycolytic capacity and reduced mitochondrial respiration in MIA-PaCa-2 cells. As a comparison, the effects of GSK-3 on MCF-7 breast cancer cells, which have mutant *PIK3CA*, were examined. KD-GSK-3β increased the resistance of MCF-7 cells to chemotherapeutic drugs and certain signal transduction inhibitors. Thus, altering the levels of GSK-3β can have dramatic effects on sensitivity to drugs and signal transduction inhibitors which may be influenced by the background of the tumor.

## 1. Introduction

Glycogen synthase kinase-3 (GSK-3) is a family of kinases consisting of GSK-3α and GSK-3β. The GSK-3 family members are highly conserved and expressed in many different types of cells and tissues [1,2,3,4]. They function as kinases and phosphorylate many proteins on serine (S) and threonine (T) residues [5,6,7,8,9]. After phosphorylation, their target proteins are often inactivated. GSK-3 kinases play key roles in many biological processes including aging, diabetes, inflammation, metabolism, neural transmission, obesity, and drug-resistance [3,4,10,11,12]. The abilities of GSK-3β to influence the drug-sensitivity of pancreatic cancer cells and breast cancer cells are the subjects of this manuscript. 

**Figure 1:**
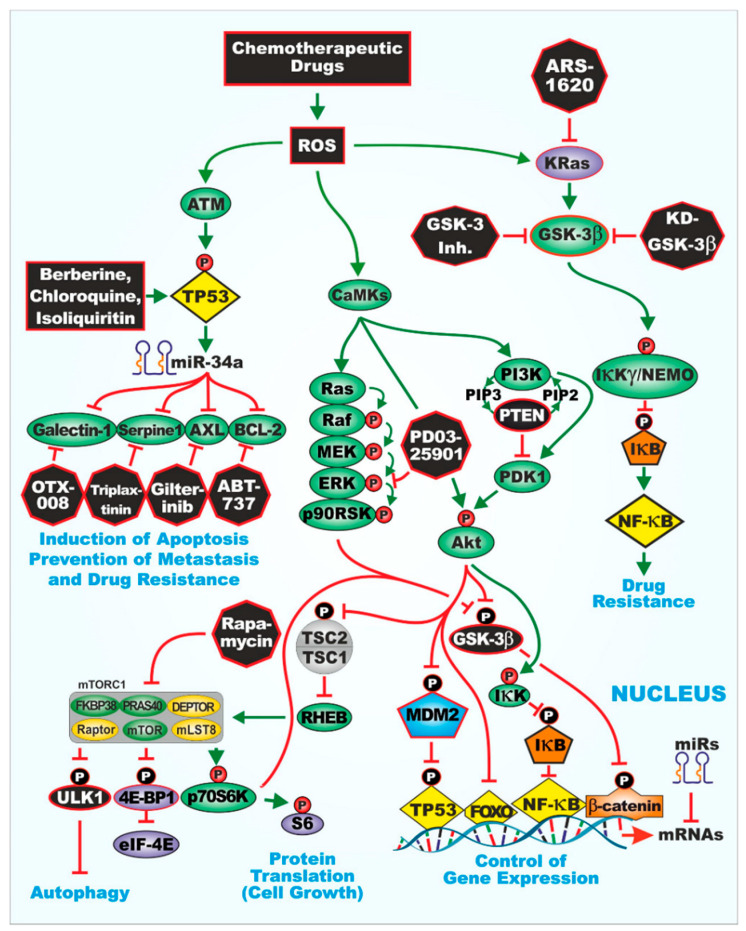
Illustration of glycogen synthase kinase-3 (GSK-3) interactions with other signaling pathways important in regulation of cell growth and sites of interaction for certain signal transduction inhibitors used in this study.

GSK-3β is often considered to function in concert with the PI3K/PTEN/AKT/mTORC1 and Wnt/β-catenin pathways [10,11,12,13,14,15,16]. An overview of the interactions with various signaling pathways and sites of certain small molecule inhibitors action is presented in Figure 1. One of the best characterized reactions involving GSK-3β is its phosphorylation at S9 by AKT, which results in its inactivation [6]. GSK-3β is then targeted for proteasomal degradation. Other kinases and phosphatases also regulate GSK-3β activity by either phosphorylation (e.g., protein kinase A (PKA), p70 ribosomal S6 kinase (p70S6K), and p90 ribosomal S6 kinase (p90^Rsk^) [5,17,18,19,20] or dephosphorylation (e.g., protein phosphorylase 1 (PP1) and protein phosphatase 2A (PP2A)) at various regulatory sites [21].

The PI3K/PTEN/AKT/mTORC1 pathway is often deregulated in human cancers due to mutations at PI3K (*PIK3CA*), *PTEN*, *AKT*, *MTOR* and tuberous sclerosis complex 2 (*TSC2*). Mutations that alter the activity of this pathway can be either activating (*PIK3CA*, *AKT*, and *MTOR*) or inactivating (*PTEN* and *TSC2*). Both types of mutations can lead to pathway activation [16,22]. In various cancers, including breast, prostate, and brain cancers, the *PTEN* tumor suppressor gene is frequently inactivated, which results in constitutive AKT activity that in turn inhibits GSK-3 activity. In pancreatic cancer, the upstream *KRAS* gene is frequently mutated, which results in the activation of the PI3K/PTEN/AKT/mTORC1 pathway [23,24]. These events can lead to the activation of AKT, which in turn, leads to inactivation of GSK-3β. Thus, one of the converging points in cancer development is phosphorylation of GSK-3 and its subsequent inactivation. Silencing of GSK-3 can lead to activation of downstream molecules such as mTOR, which promote cell growth. Therefore, in many cancers with aberrant AKT activity, subsequent suppression of GSK-3 may prevent its normal negative regulatory effects on cellular proliferation. This may contribute to altered sensitivity to chemotherapeutic drugs, signal transduction inhibitors and nutraceuticals and the cancer cells can proliferate in an uncontrolled fashion. 

An example of this dysregulation is the effects of GSK-3 on the mechanistic target of rapamycin (mTORC1) activity. mTORC1 activity is important in cell growth and autophagy. mTORC1 activity is blocked by the immunosuppressive drug rapamycin and PI3K/mTOR dual inhibitors [14,15]. Both types of drugs are used to treat patients with various diseases. AMP-activated protein kinase (AMPK) can phosphorylate TSC2 at S1345, GSK-3β can then phosphorylate the TSC2 protein at T1329, S1333, S1337, and S1345. TSC2 activity is suppressed and results in inhibition of downstream Ras homolog enriched in brain (RHEB) which can normally activate mTORC1. In this scenario, mTORC1 is suppressed by GSK-3 and its ability to aid in the translation of growth-regulatory mRNAs which have unique 3′ends, which are normally difficult to translate, is suppressed [10,14,15]. 

GSK-3β can phosphorylate other proteins important in protein translation such as p70S6K [17,18] and eukaryotic translation initiation factor 4E (eIF4E)-binding protein 1 (4E-BP1) [20]. Phosphorylation of p70S6K at S371 by GSK-3β positively regulates p70S6K activity, while phosphorylation of 4E-BP1 at T37/T46 by GSK-3β results in its inhibition [20]. Certain of these GSK-3β phosphorylation events are phosphorylation site specific as phosphorylation of p70S6K at T389 results in activation of TSC2 and inactivation of p70S6K [19]. These observations serve to document the fine details of GSK-3β phosphorylation, even on the same protein.

GSK-3β plays roles in the sensitivity to chemotherapeutic drugs, signal transduction inhibitors, nutraceuticals, and other small molecule inhibitors [3,4,25,26,27,28,29,30,31,32]. GSK-3β plays important roles by phosphorylating key proteins in the WNT/β-catenin complex (e.g., β-catenin, APC, Axin, LPR5/6) [10]. This complex is involved in the epithelial-mesenchymal transition (EMT), which is critical for normal growth and differentiation as well as cancer progression [10,33]. The roles of GSK-3β in cancer may differ according to the cancer type and the genetic mutations present [3,4]. Suppression of GSK-3 can lead to enhanced WNT/β-catenin activity. Some proteins such as β-catenin may have mutations in the GSK-3β phosphorylation sites in certain cancer cells, which prevent GSK-3β-dependent phosphorylation and lead to constitutive activation of the Wnt/β-catenin pathway and contribute to cancer development. If β-catenin activity is increased due to the inability of GSK-3β to phosphorylate it and inactivate it, increased proliferation and drug resistance may occur. Additional studies have shown that GSK-3β may exert positive effects on cell proliferation and the GSK-3β protein is overexpressed in certain tumor types, including colon, liver, ovarian, and pancreatic cells [34,35,36]. There are key interactions between NF-κB and GSK-3β, which result in important loops that stimulate the growth of certain cancers. 

We have shown that inhibition of GSK-3β activity may increase the drug-resistance of breast cancer cells to certain chemotherapeutic drugs [37]. This may result in the activation of the Wnt/β-catenin pathway and cell growth, differentiation, and drug resistance [10]. Moreover, inactivation of GSK-3 prevents phosphorylation of the transcription factor Slug, which results in promotion of EMT and metastasis [3,4,38,39].

Pancreatic cancer is often diagnosed late in the development of the disease resulting in poor treatment outcomes. Approximately 80% of pancreatic cancers are pancreatic ductal carcinomas (PDAC) [40]. Surgery is the most common approach for PDAC treatment, although patients may also be treated with chemotherapeutic drugs (e.g., 5-fluorouracil (5FU), gemcitabine, cisplatin, oxaliplatin, paclitaxel, irinotecan, and others). However, these chemotherapeutic drug treatments are usually not curative and by the time the PDAC is diagnosed it is often advanced and nearly impossible to treat [41]. The survival period after treatment of PDAC patients is quite short, usually less than a year with a 5-year survival rate of <5% [5,42]. Clearly novel, more effective approaches to treat PDAC are essential for improved treatment. PDAC patients have mutations at *KRAS*, *TP53*, and various other oncogenes and tumor suppressor genes [43]. Inhibitors specific for mutant KRas or reactivation of WT-TP53 activity have proven difficult to develop. GSK-3 inhibitors inhibit the growth of certain PDAC tumors. There are interactions between Kras, GSK-3, NF-κB, and TP53 [44,45]. Thus, an alternative approach may be to treat PDAC patients, who are dependent on mutant KRas, with GSK-3 inhibitors [36,46,47]. 

In the following studies, we investigated the effects of GSK-3β expression on the sensitivity of the MIA-PaCa-2 pancreatic cancer and the MCF-7 breast cancer cell lines to various chemotherapeutic drugs, signal transduction inhibitors, and nutraceuticals. The MIA-PaCa-2 PDAC cell line is a good model to examine the effects of GSK-3β on PDAC drug sensitivity as it contains mutations at *KRAS* and *TP53* [48], two of the most frequently mutated genes in PDAC. Expression of ectopic WT-GSK-3β often increased the resistance of MIA-PaCa-2 cells to various chemotherapeutic drugs including: 5FU, paclitaxel, cisplatin, docetaxel, irinotecan, doxorubicin, daunorubicin, and mitoxantrone in comparison to cells transfected with either KD-GSK-3β or the empty vector pLXSN. In contrast, cells transfected with WT-GSK-3β were more sensitivity to an inhibitor that targets BCL2 and BCLXL than cells transfected with KD-GSK-3β or pLXSN. WT-GSK-3β also had increased metabolic and glycolytic activities in comparison to cells transfected with KD-GSK-3β. We also examined the effects of introduction WT-GSK-3β and KD-GSK-3β on the sensitivity of MCF-7 breast cancer cells to chemotherapeutic drugs, signal transduction inhibitors and a nutraceutical. In contrast to the results observed in MIA-PaCa-2 PDAC cells, where WT-GSK-3β acted as a tumor promoter and KD-GSK-3β functioned as a tumor suppressor, in MCF-7 breast cancer cells, KD-GSK-3β functioned as a tumor promoter and WT-GSK-3β functioned as a tumor suppressor. These results document the complexity of GSK-3β in regulation of therapeutic sensitivity which is likely dependent of the presence of different mutations in various cell types.

## 2. Materials and Methods

### 2.1. Cell Culture and Chemotherapeutic Drugs, Signal Transduction Inhibitors and Nutraceuticals

MIA-PaCa-2 PDAC cells (ATCC CRM-CRL-1420) were obtained from the American Type Culture Collection (ATCC) (Manassas, VA, USA). The cells were recovered from a 65-year old Caucasian male PDAC patient [48]. MIA-PaCa-2 cells were cultured as described [49]. 

MCF-7 breast cancer cells (ATCC^®^ HTB-22^TM^) were obtained from the ATCC. They were derived from a metastatic site pleural effusion of a breast carcinoma from a 69-year old female [50]. MCF-7 cells were cultured as described [37]. Chemotherapeutic drugs, signal transduction inhibitors and nutraceuticals were obtained from either Sigma-Aldrich (Saint Louis, MO, USA) or Selleck Chemicals (Houston, TX, USA).

### 2.2. WT-GSK-3β, KD-GSK-3β and pLXSN Plasmids

Plasmid DNAs encoding WT-GSK-3β and KD-GSK-3β [7,51] were generously provided by Dr. James Woodgett (University of Toronto, Toronto, ON, Canada). KD-GSK-3β differs from WT-GSK-3β by a substitution of methionine and alanine for lysine at positions 85 and 86, respectively. The KD-GSK-3β inhibits the activity of endogenous GSK-3β [35]. The WT-GSK-3β and KD-GSK-3β expression vectors also contain the gene encoding resistance to geneticin (G418). pLXSN is an empty retroviral vector which contains the gene encoding resistance to G418. pLXSN was generously provided by A. Dusty Miller, Fred Hutchinson Cancer Center, Seattle, WA, USA) [52]. 

### 2.3. Transfection of MIA-PaCa-2 and MCF-7 Cells with GSK-3β Constructs

Next, 5 × 10^5^ MIA-PaCa-2 or MCF-7 cells were plated into 6-well cell culture plates (BD Biosciences, San Jose, CA, USA) and transfected with the various plasmids as described [37]. Cells were transfected the WT-GSK-3β and KD-GSK-3β DNAs as described [37] with Lipofectin (Invitrogen) as described by the manufacturer. The generation of MIA-PaCa-2 + pLXSN cells were previously described [49]. Further, 48 h after transfection, a selection medium (DMEM + 5% FBS or RPMI-1640 + 5% FBS + 2 mg/mL G418 (Geneticin) (Invitrogen) was added to isolate stably transfected cells. Cells were fed with a fresh selection medium every three days. Mock transfections were also performed and did not generate viable colonies in the presence of selection medium. 

### 2.4. Cell Proliferation Assays in the Presence of Chemotherapeutic Drugs, Signal Transduction Inhibitors, and Nutraceuticals

MIA-PaCa-2 + WT-GSK-3β, MIA-PaCa-2 + KD-GSK-3β, MIA-PaCa-2 + pLXSN, MCF-7 + WT-GSK-3β, MCF-7 + KD-GSK-3β, and MCF-7 + pLXSN cells were seeded in 96-well cell culture plates (BD Biosciences, San Jose, CA) at a density of 5000 cells/well in 100 μL of phenol red free RPMI-1640 containing 1% FBS as described [37,49]. The treatment medium was prepared by performing 10 two-fold serial dilutions to create a range of 11 concentrations of the different drugs, signal transduction inhibitors, and nutraceuticals. After 72 h of treatment (four days after seeding), the amount of 3-(4,5-dimethylthiazol-2-yl)-2,5-diphenyl-2*H*-tetrazolium bromide (MTT) (Sigma-Aldrich) reduction in each well was quantified as described [37,49]. The absorbance at 570 nM was determined with a FL600 microplate fluorescence reader (Bio-Tek Instruments; Winooski, VT, USA) as described [37,49]. The mean and corresponding standard deviation of normalized adjusted absorbance was calculated from three replicate wells for each drug concentration. The inhibitory concentration of 50% (IC_50_) is defined in this context as the concentration of the drug that causes MIA-PaCa-2 or MCF-7 cells to proliferate at a rate that is half as rapid as cells incubated in the absence of the drug.

The drug concentrations used in human therapy are usually much higher than those used to treat tissue culture cells. This is because drug delivery to humans has many more factors which restrict the effects of the drugs in various human organs (e.g., liver) than in tissue culture cells in vitro. None of the drugs/nutraceuticals in our studies exceeded the drug concentrations that are used clinically. Thus, our studies provide a model for the effects of these drugs in vitro.

### 2.5. Colony-Formation Assays

MIA-PaCa-2 + WT-GSK-3β, MIA-PaCa-2 + KD-GSK-3β, and MIA-PaCa-2 + pLXSN cells were collected and seeded in 6-well cell culture plates at a density of 500 cells/well in 2 mL of DMEM + 5% FBS for each well (three replicate wells for each condition) as described [37,49]. Next, 24 h after seeding, plates were then treated with different concentrations of 5FU, gemcitabine, doxorubicin, tideglusib, metformin, or berberine in 2 mL of DMEM + 5% FBS for each well and incubated for three weeks at 37 °C as described [37,49]. At the end of the three-week treatment period, fixed cells were incubated in Giemsa stain (Sigma) for 5 min at room temperature as described [37,49]. Colonies consisted of at least 50 cells and the number of colonies present in each well was counted. The mean number of colonies and corresponding standard deviation was calculated from three replicate wells for each condition. The colony formation abilities were determined three times for each cell type and each treatment condition. Statistical significance was calculated using the GraphPad QuickCalcs software (San Diego, CA, USA) using an unpaired *t* test with a 95% confidence interval. 

### 2.6. Real-Time Cell Metabolic Analysis

Mitochondrial activity was measured by performing mitochondrial stress tests and glycolysis stress tests with the Seahorse instrument (Agilent, Santa Clara, CA, USA) as described [53]. Briefly, exponentially growing cells in tissue culture flasks were washed with phosphate buffered saline and then treated with 1X trypsin (Life Technologies) for 5 min. The cells were then briefly centrifuged, and the cell numbers were determined on an automatic cell counter after staining with trypan blue. 100,000 cells of each cell type in a volume of 200 microliters of standard tissue culture medium was then added to 5 wells for each cell type on a Seahorse 24 well plate. The cells were allowed to adhere to the plate for 1 h at 37 °C. Then, the 24 well plate was placed in the Seahorse instrument and the various agents were added at the indicated time periods on the graphs (e.g., glucose, oligomycin, 2-deoxyglucose, BAM15, rotenone, and antimycin A). After the Seahorse experiments were performed, the actual protein concentrations in each well were determined and standardized. Briefly, the cells were lysed using the RIPA buffer, and total protein content of each well was determined using the Pierce BCA Protein Assay Kit (Cat# 23227, ThermoFisher Scientific, (Waltham, MA, USA). Then the oxygen consumption rate (OCR) or the extracellular acidification rate (ECAR) value of each well was divided by the total protein concentration of that well. The statistical significance was determined by the Mann–Whitney test with Graph Pad software (San Diego, CA, USA).

## 3. Results

To determine the effects of GSK-3β on the sensitivity of PDAC cancer cells to chemotherapy, targeted therapy and nutraceuticals, MIA-PaCa-2 PDAC cells were transfected with wild-type (WT), kinase dead (KD) forms of GSK-3β [7,51]. As controls for these experiments, we also examined the effects of pLXSN, which is an empty vector encoding NeoR [52]. The effects of various chemotherapeutic drugs on the drug sensitivity of control empty vector pLXSN and untransfected cells (parental lines) were examined in some cases. The sensitivities of the empty vector pLXSN and untransfected cells were similar. 

### 3.1. Effects of GSK-3β on Sensitivities of PDAC Cells to Chemotherapeutic Drugs Used to Treat PDAC Patients

**Figure 2:**
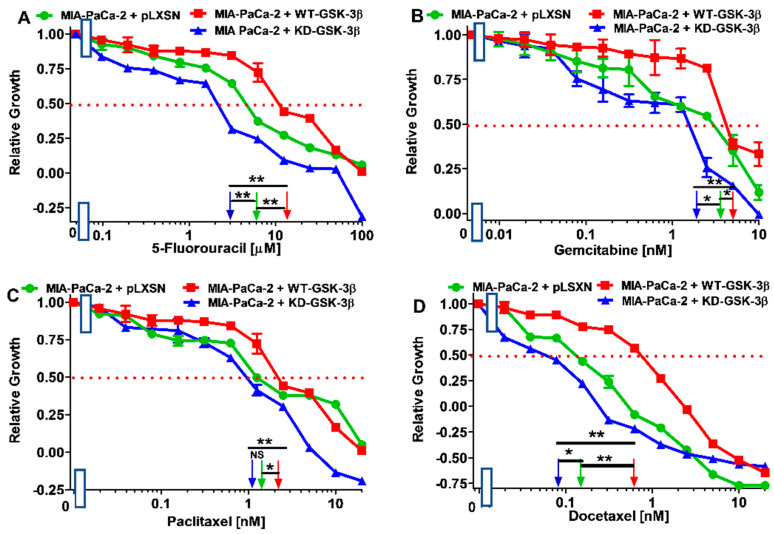
Effects of chemotherapeutic drugs on the growth of MIA-PaCa-2 + WT-GSK-3β, MIA-PaCa-2 + KD-GSK-3β, and MIA-PaCa-2 + pLXSN cells. The effects of 5FU (Panel (**A**)), gemcitabine (Panel (**B**)), paclitaxel (Panel (**C**)), and docetaxel (Panel (**D**)) on MIA-PaCa-2 + WT-GSK-3β (solid red boxes), MIA-PaCa-2 + KD-GSK-3β (solid blue triangles), and MIA-PaCa-2 + pLXSN cells (solid green circles) were examined using MTT analysis. The MIA-PaCa-2 + WT-GSK-3β, MIA-PaCa-2 + KD-GSK-3β, and MIA-PaCa-2 + pLXSN cells in each panel were all examined at the same time period. These experiments were repeated 6 times and similar results were obtained. Statistical analyses were performed via the Student’s *T* test on the means and standard deviations of various treatment groups. ** *p* < 0.005, * *p* < 0.05 and NS = not statistically significant.

**Figure 3:**
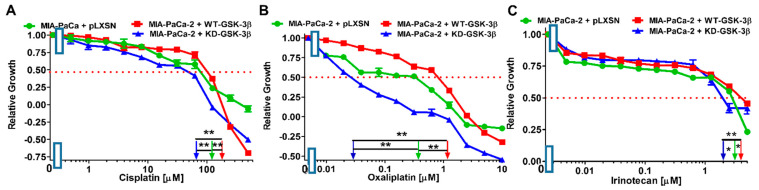
Effects of chemotherapeutic drugs on the growth of MIA-PaCa-2 + WT-GSK-3β, MIA-PaCa-2 + KD-GSK-3β, and MIA-PaCa-2 + pLXSN cells. The effects of cisplatin (Panel (**A**)), oxaliplatin (Panel (**B**)), and irinotecan (Panel (**C**)) on MIA-PaCa-2 + WT-GSK-3β (solid red boxes), MIA-PaCa-2 + KD-GSK-3β (solid blue triangles) and MIA-PaCa-2 + pLXSN Cells (solid green circles) were examined by MTT analysis. The MIA-PaCa-2 + WT-GSK-3β, MIA-PaCa-2 + KD-GSK-3β and MIA-PaCa-2 + pLXSN cells in each panel were all examined at the same time period. These experiments were repeated 6 times and similar results were obtained. Statistical analyses were performed via the Student’s *T* test on the means and standard deviations of various treatment groups. ** *p* < 0.005 and * *p* < 0.05.

The effects of WT-GSK-3β, KD-GSK-3β, and pLXSN on the sensitivity to MIA-PaCa-2 cells to chemotherapeutic drugs were used to treat PDAC are presented in Figure 2 and Figure 3, and Table 1. MIA-PaCa-2 cells were used in the following study as they represent an in vitro model for pancreatic cancer. MIA-PaCa-2 cells have an activating mutation in *KRAS* and a gain of function mutation at *TP53*, as well as some other mutations important in pancreatic cancer cells. They are also estrogen-receptor (ER) positive and metastatic [48]. These characteristics are often present in pancreatic cancer. Other pancreatic cancer cell lines lack some of these properties. 

Introduction of WT-GSK-3β into MIA-PaCa-2 cells resulted in an increase of the IC_50_ values, i.e., decrease of sensitivity, of the cells to all the tested chemotherapeutic drugs used to treat PDAC patients, as compared to the control. The greatest decrease of sensitivity was observed with docetaxel and oxaliplatin.

In turn, introduction of KD-GSK-3β into these cells resulted in about 2-fold decreases of the IC_50_ values, i.e., increase of sensitivity, of the cells to almost all the tested chemotherapeutics. The exception was oxaliplatin to which the sensitivity increased over 13 times.

Comparison of the effects of transfection of the cells with WT-GSK-3β and KD-GSK-3β, the most dramatic decreases in sensitivities to chemotherapeutic drugs were observed after introduction of the WT-GSK-3β for oxaliplatin (almost 37-fold) and docetaxel (7.5-fold). For the rest of the tested chemotherapeutics, the sensitivities decreased about 2-4-fold. 

### 3.2. Effects of WT-GSK-3β and KD-GSK-3β on the Sensitivities of MIA-PaCa-2 Cells to Chemotherapeutic Drugs Used to Treat Patients with Other Types of Cancer

**Figure 4:**
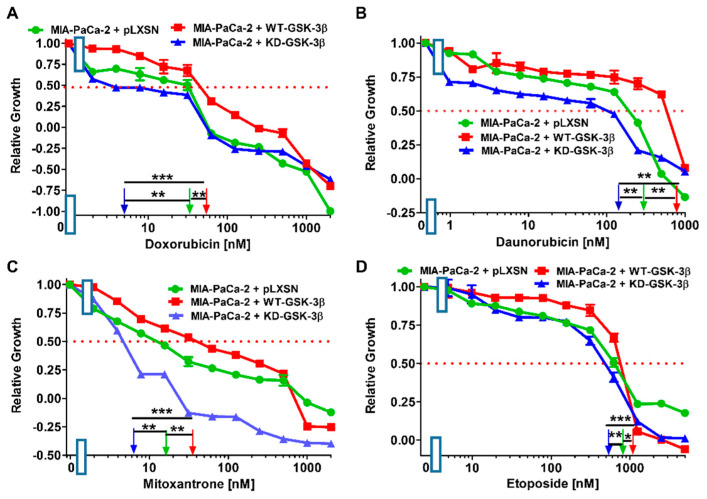
Effects of chemotherapeutic drugs on the growth of MIA-PaCa-2 + WT-GSK-3β, MIA-PaCa-2 + KD-GSK-3β and MIA-PaCa-2 + pLXSN cells. The effects of doxorubicin (Panel (**A**)), daunorubicin (Panel (**B**)), mitoxantrone (Panel (**C**)), and etoposide (Panel (**D**)) on MIA-PaCa-2 + WT-GSK-3β (solid red boxes), MIA-PaCa-2 + KD-GSK-3β (solid blue triangles) and MIA-PaCa-2 + pLXSN Cells (solid green circles) were examined by MTT analysis. The MIA-PaCa-2 + WT-GSK-3β, MIA-PaCa-2 + KD-GSK-3β, and MIA-PaCa-2 + pLXSN cells in each panel were all examined at the same time period. These experiments were repeated 4 times and similar results were obtained. Statistical analyses were performed via the Student’s *T* test on the means and standard deviations of various treatment groups. *** *p* < 0.0001, ** *p* < 0.005, and * *p* < 0.05.

Next we examined the effects of introduction of WT- and KD-GSK-3β on the chemosensitivities of other drugs frequently used to treat other types of cancer patients as this may provide additional information important for determining the effects of GSK-3β on chemotherapeutic drug-resistance. Results of these experiments are presented in Figure 4 and Table 1. As in the case of chemotherapeutics used for pancreatic cancer treatment, introduction of WT-GSK-3β into MIA-PaCa-2 cells resulted in an increase of the IC_50_ values, i.e., decrease of sensitivity, of the cells to all the tested compounds.

### 3.3. Effects of WT-GSK-3β and KD-GSK-3β on Sensitivities to GSK-3β Inhibitors

**Figure 5:**
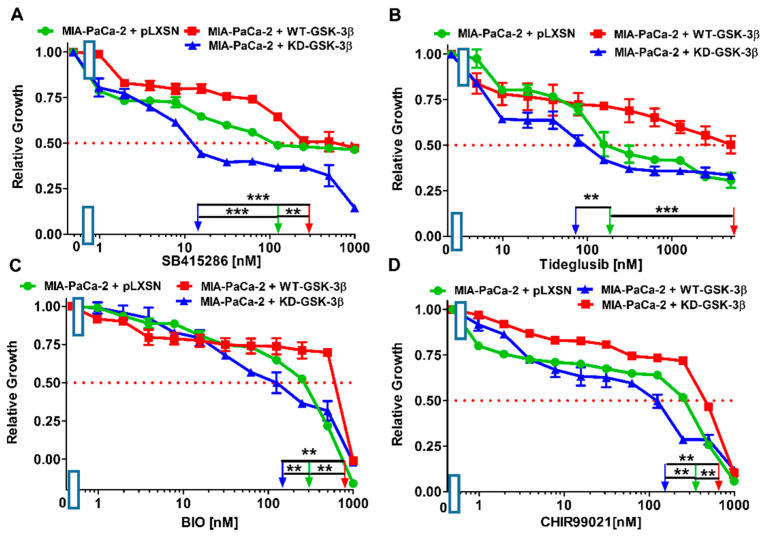
Effects of GSK-3 inhibitors on the growth of MIA-PaCa-2 + WT-GSK-3β, MIA-PaCa-2 + KD-GSK-3β and MIA-PaCa-2 + pLXSN cells. The effects of SB415286 (Panel (**A**)), Tideglusib (Panel (**B**)), BIO (Panel (**C**)) and CHIR99021 (Panel (**D**)) on MIA-PaCa-2 + WT-GSK-3β (solid red boxes), MIA-PaCa-2 + KD-GSK-3β (solid blue triangles) and MIA-PaCa-2 + pLXSN Cells (solid green circles) were examined by MTT analysis. The MIA-PaCa-2 + WT-GSK-3β, MIA-PaCa-2 + KD-GSK-3β and MIA-PaCa-2 + pLXSN cells in each panel were all examined at the same time period. These experiments were repeated 3 times and similar results were obtained. Statistical analyses were performed via the Student’s *T* test on the means and standard deviations of various treatment groups. *** *p* < 0.0001 and ** *p* < 0.005.

As stated previously, GSK-3 plays various roles in cancer, including tumor promoter and tumor suppressor activities. Inhibition of GSK-3 activity has been proposed for the treatment of PDAC [54,55]. Thus, the effects of four structurally diverse GSK-3 inhibitors [3] were examined: SB415286, tideglusib, 6-bromoindirubin-30-oxime (BIO), and CHIR99021. Results of these experiments are presented in Figure 5 and summarized in Table 1.

KD-GSK-3β reduced the resistance of MIA-PaCa-2 cells to the GSK-3 inhibitors 8-fold to SB415286 and about 2-fold for the remaining three compounds, compared with the cells transfected with the empty vector.

Direct comparison of the effects of the introduction of WT-GSK-3β and KD-GSK-3β into MIA-PaCa-2 cells revealed augmented resistance to tideglusib over 70-fold and to SB415286 20-fold. Thus, the results indicated an inverse correlation between WT-GSK-3β and vulnerability of MIA-PaCa-2 cells to chemotherapy and GSK-3 inhibitors. 

### 3.4. Effects of WT-GSK-3β and KD-GSK-3β on Sensitivities of MIA-PaCa-2 to Signal Trans-Duction Inhibitors Targeting Other Pathways

**Figure 6:**
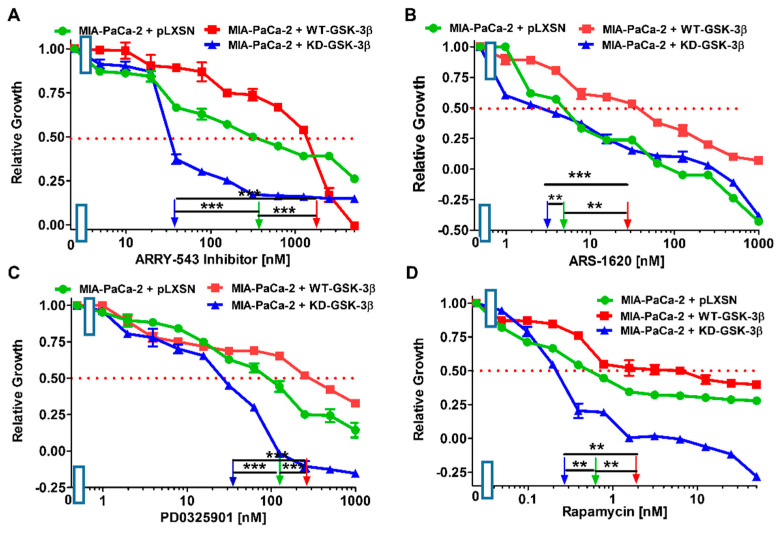
Effects of signal transduction inhibitors on the growth of MIA-PaCa-2 + WT-GSK-3β, MIA-PaCa-2 + KD-GSK-3β and MIA-PaCa-2 + pLXSN cells. The effects of the pan EGFR ARRY-543 inhibitor (Panel (**A**)), the KRAS ARS-1620 inhibitor (Panel (**B**)), the MEK1 PD0325901 inhibitor (Panel (**C**)), and the mTORC1 blocker rapamycin (Panel (**D**)) on MIA-PaCa-2 + WT-GSK-3β (solid red boxes), MIA-PaCa-2 + KD-GSK-3β (solid blue triangles) and, MIA-PaCa-2 + pLXSN Cells (solid green circles) were examined by MTT analysis. The MIA-PaCa-2 + WT-GSK-3β, MIA-PaCa-2 + KD-GSK-3β and MIA-PaCa-2 + pLXSN cells in each panel were all examined at the same time period. These experiments were repeated 4 times and similar results were obtained. Statistical analyses were performed via the Student’s *T* test on the means and standard deviations of various treatment groups. *** *p* < 0.0001, and ** *p* < 0.005.

There are mutations in various genes (e.g., *KRAS*, *TP53*, *PIK3CA*, *CTNNB1* (β-catenin), *AXIN*, and others) that effect the ability of GSK-3β to phosphorylate its substrates. These mutations may change the sensitivity to signal transduction pathways inhibitors. There are also interactions between GSK-3, NF-κB, and TP53, which alter the sensitivity to signal transduction inhibitors [44,56,57,58,59,60,61,62,63]. GSK-3 can affect the activity of NF-κB by phosphorylation of the IKKγ/NEMO substrate [61]. NF-κB is a critical transcription factor involved in inflammation, cancer and many biological processes. The effects of introduction of WT and KD-GSK-3β on the sensitivities of MIA-PaCa-2 cells to various signal transduction pathway inhibitors were examined (Figure 6, Table 1). 

ARRY-543 is a pan-EGFR inhibitor [63]. EGFR and HER2 are important receptors that play critical roles in many cancers [23]. MIA-PaCa-2 cells express both EGFR and HER2 [62]. MIA-PaCa-2 cells have a mutant *KRAS* gene [48]. They have the G12C *KRAS* mutation on both alleles [32]. ARS-1620 is an inhibitor which suppresses cells with this mutation [64]. Downstream of KRAS are the Raf/MEK/ERK and the PI3K/PTEN/Akt/mTORC1 pathways. PD0325901 is a MEK1 inhibitor and rapamycin is a mTORC1 blocker [13,14,15,16]. 

Introduction of WT-GSK-3β into MIA-PaCa-2 cells resulted in increases of the IC_50_ values (decrease of sensitivity) to all the tested inhibitors, as compared to MIA-PaCa-2 cells transfected with the empty vector. In this context, it was not unexpected that the introduction of kinase-dead GSK-3β into these cells increased their sensitivities to the tested inhibitors of diverse pathways.

Direct comparison of the effects of the introduction of WT-GSK-3β and KD-GSK-3β to MIA-PaCa-2 cells revealed that increased expression of WT-GSK-3β augmented the resistance of the cells to EGFR pathway inhibitor ARRY-543 50-fold. The increases in resistance to remaining pathways inhibitors were lower but still significant. 

### 3.5. Effects of WT-GSK-3β and KD-GSK-3β on Sensitivity to the BCL2/BCLXL ABT-737 Inhibitor

**Figure 7:**
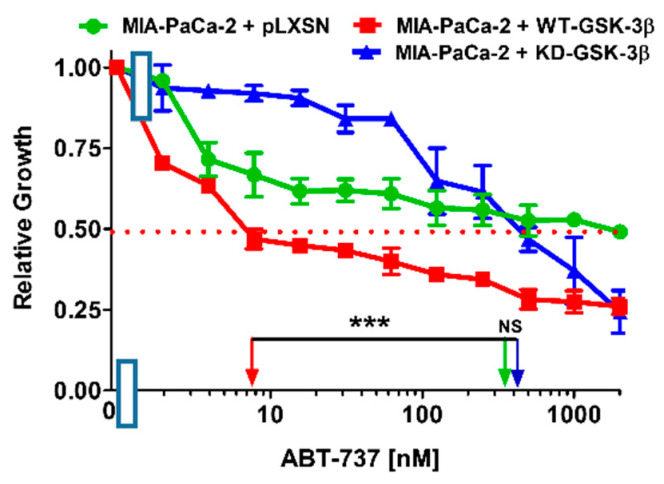
Effects of the BCL2/BCLXL ABT-737 inhibitor on the growth of MIA-PaCa-2 + WT-GSK-3β, MIA-PaCa-2 + KD-GSK-3β and MIA-PaCa-2 + pLXSN cells. The effects of the BCL2/BCLXL inhibitor ABT-737 on MIA-PaCa-2 + WT-GSK-3β (solid red boxes), MIA-PaCa-2 + KD-GSK-3β (solid blue triangles) and MIA-PaCa-2 + pLXSN Cells (solid green circles) were examined using MTT analysis. The MIA-PaCa-2 + WT-GSK-3β, MIA-PaCa-2 + KD-GSK-3β, and MIA-PaCa-2 + pLXSN cells in each panel were all examined at the same time period. These experiments were repeated 4 times and similar results were obtained. Statistical analyses were performed via the Student’s *T* test on the means and standard deviations of various treatment groups. *** *p* < 0.0001, NS = not statistically significant.

BCL2 and BCLXL play critical roles in apoptosis and cancer development and they can be regulated by GSK-3 [3]. The IC_50_ of the BCL2/BCLXL ABT-737 inhibitor in MIA-PaCa-2 + pLXSN cells was approximately 350 nM (Figure 7). Introduction of WT-GSK-3β into these cells reduced the IC_50_ to approximately 7 nM, i.e., 50-fold lower than that observed in MIA-PaCa-2 + pLXSN cells. Upon introduction of KD-GSK-3β, the IC_50_ for ABT-737 was approximately 350 nM, the same as in MIA-PaCa-2 + pLXSN cells. The IC_50_ for ABT-737 was approximately 50-fold lower in MIA-PaCa-2 + WT-GSK-3β than in MIA-PaCa-2 + KD-GSK-3β cells (Figure 7).

### 3.6. Effects of WT-GSK-3β and KD-GSK-3β on Drugs Used to Treat Diabetes, Malaria, and the Nutraceutical Berberine 

Commonly used drugs such as metformin and chloroquine were originally developed to treat diseases such as type II diabetes and malaria, respectively. Recently, these drugs have been shown to have anti-cancer properties [3,11,12,49]. 

**Figure 8:**
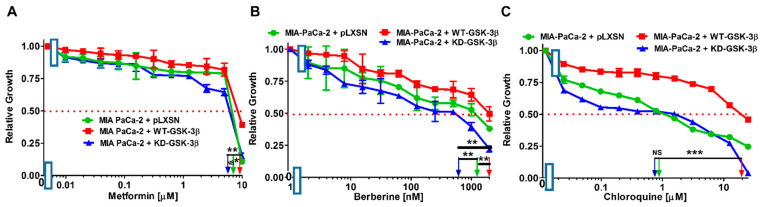
Effects of drugs used to treat diabetes, malaria and the nutraceutical berberine on the growth of MIA-PaCa-2 + WT-GSK-3β, MIA-PaCa-2 + KD-GSK-3β, and MIA-PaCa-2 + pLXSN cells. The effects of the AMPK activator metformin (Panel (**A**)), the nutraceutical berberine (Panel (**B**)), and the anti-malarial drug chloroquine (Panel (**C**)) on MIA-PaCa-2 + WT-GSK-3β (solid red boxes), MIA-PaCa-2 + KD-GSK-3β (solid blue triangles) and MIA-PaCa-2 + pLXSN Cells (solid green circles) were examined by MTT analysis. The MIA-PaCa-2 + WT-GSK-3β, MIA-PaCa-2 + KD-GSK-3β, and MIA-PaCa-2 + pLXSN cells in each panel were all examined at the same time period. These experiments were repeated 4 times and similar results were obtained. Statistical analyses were performed by the Student *T* test on the means and standard deviations of various treatment groups. *** *p* < 0.0001, ** *p* < 0.005, * *p* < 0.05 and NS = not statistically significant

Introduction of WT-GSK-3β into MIA-PaCa-2 cells resulted in about 1.3-fold increase of IC_50_ for metformin compared to MIA-PaCa-2 + pLXSN cells. Upon introduction of KD-GSK-3β, the IC_50_ for metformin was decreased approximately by the same amount as in MIA-PaCa-2 + pLXSN cells. The IC_50_ for metformin was approximately 1.5-fold higher in MIA-PaCa-2 + WT-GSK-3β than in MIA-PaCa-2 + KD-GSK-3β cells (Figure 8A, Table 1).

Berberine is used in traditional medicine to treat diabetes and other diseases [49]. Berberine can inhibit cell growth and induce apoptosis in many cells. It can also induce TP53. Berberine and metformin share some similar effects [49].

Introduction of WT-GSK-3β into MIA-PaCa-2 cells resulted in a berberine IC_50_ 1.7-fold higher than that observed in MIA-PaCa-2 + pLXSN cells. In turn, upon the introduction of KD-GSK-3β, the IC_50_ for berberine was about 2-fold reduced. Direct comparison of the effects of the introduction of WT-GSK-3β and KD-GSK-3β to MIA-PaCa-2 cells revealed that increased expression of WT-GSK-3β elevated the resistance to berberine over 3-fold (Figure 8B, Table 1).

The IC_50_ of chloroquine in MIA-PaCa-2 + pLXSN cells was similar as that observed in MIA-PaCa-2 + KD-GSK-3β cells, and it was approximately 22-fold lower than in MIA-PaCa-2 + WT-GSK-3β (Figure 8C, Table 1), which means that introduction of WT-GSK-3β increased resistance of the cells to this anti-malarial drug.

Thus, WT-GSK-3β increased the resistance of MIA-PaCa-2 to some drugs used to treat diabetes and malaria.

### 3.7. Effects of WT-GSK-3β and KD-GSK-3β on the Sensitivities to Drugs Used to Suppress Cancer Progression and Metastasis

**Figure 9:**
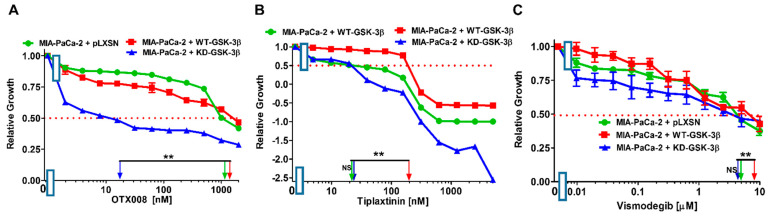
Effects of inhibitors which target events involved in metastasis on the growth of MIA-PaCa-2 + WT-GSK-3β, MIA-PaCa-2 + KD-GSK-3β, and MIA-PaCa-2 + pLXSN cells. The effects of the galectin-1 inhibitor OTX008 (Panel (**A**)), the serpine-1 inhibitor tiplaxtinin (Panel (**B**)) and the Hh pathway inhibitor vismodegib (Panel (**C**)) on MIA-PaCa-2 + WT-GSK-3β (solid red boxes), MIA-PaCa-2 + KD-GSK-3β (solid blue triangles), and MIA-PaCa-2 + pLXSN Cells (solid green circles) were examined via MTT analysis. The MIA-PaCa-2 + WT-GSK-3β, MIA-PaCa-2 + KD-GSK-3β, and MIA-PaCa-2 + pLXSN cells in each panel were all examined at the same time period. These experiments were repeated 4 times and similar results were obtained. Statistical analyses were performed by the Student *T* test on the means and standard deviations of various treatment groups. ** *p* < 0.005, and NS = not statistically significant.

Galectin-1 has been implicated in the metastasis of many cancers, including PDAC [65]. OTX008 is a galectin-1 inhibitor [66]. Introduction of WT-GSK-3β into MIA-PaCa-2 cells slightly increased the IC_50_ for this compound, as compared to control cells. Introduction of KD-GSK-3β had a more pronounced effect—it decreased the IC_50_ almost 60-fold. Thus, in comparison to KD-GSK-3β-expressing cells, introduction of WT-GSK-3β resulted in nearly 70-fold decrease in sensitivity of MIA-PaCa-2 cells to OTX008 (Figure 9A, Table 1).

Serpine-1, also known as plasminogen activator inhibitor-1 (PAI-1), is a gene implicated in the progression of many cancers [67]. It is regulated by TP53 in many cells. We have previously shown that serpine-1 is regulated by TP53/miR-34a in MIA-PaCa-2 cells as well as primary PDAC patient samples [68]. Tiplaxtinin is a serpine-1 inhibitor [69]. The IC_50_ of tiplaxtinin in MIA-PaCa-2 + pLXSN cells was approximately 20 nM (Figure 8C), which is similar to MIA-PaCa-2 + KD-GSK-3β cells. Introduction of WT-GSK-3β into MIA-PaCa-2 cells resulted in a 10-fold increase of this value (Figure 9B, Table 1).

The hedgehog (Hh) pathway has been implicated in the progression of PDAC and other cancer types [70]. GSK-3 interacts with certain components of the Hh pathway. Vismodegib (Erivedge) inhibits the Hh pathway as it is an antagonist of the smoothened receptor (SMO), which is a key regulator of the pathway. Its effects in combination with gemcitabine and nab-paclitaxel have examined in phase 2 studies with PDAC patients [71,72]. 

**Figure 10:**
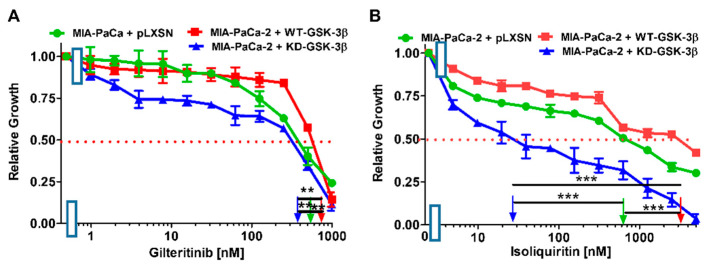
Effects of inhibitors which target events involved in metastasis on the growth of MIA-PaCa-2 + WT-GSK-3β, MIA-PaCa-2 + KD-GSK-3β, and MIA-PaCa-2 + pLXSN cells. The effects of the ALK/AXL/FLT3 gilteritinib (Panel (**A**)) and the nutraceutical isoliquiritin (Panel (**B**)) on MIA-PaCa-2 + WT-GSK-3β (solid red boxes), MIA-PaCa-2 + KD-GSK-3β (solid blue *triangles*), and MIA-PaCa-2 + pLXSN Cells (solid green circles) were examined via MTT analysis. The MIA-PaCa-2 + WT-GSK-3β, MIA-PaCa-2 + KD-GSK-3β and MIA-PaCa-2 + pLXSN cells in each panel *were* all examined at the same time period. These experiments were repeated 4 times and similar results were obtained. Statistical analyses were performed by the Student *T* test on the means and standard deviations of various treatment groups. *** *p* < 0.0001, ** *p* < 0.005, and NS = not statistically significant.

The tyrosine kinase AXL has been implicated in cancer metastasis [73]. AXL/ALK/FLT3 inhibitors (e.g., gilteritinib) have shown promise as anti-cancer agents [74]. The IC_50_ of gilteritinib in MIA-PaCa-2 + pLXSN cells was approximately 500 nM and increased about 1.3-times in cells overexpressing WT-GSK-3β (Figure 10A, Table 1). In contrast, the gilteritinib IC_50_ in MIA-PaCa-2 + KD-GSK-3β cells was approximately 400 nM, 1.4- and 1.8-fold lower than that that observed in MIA-PaCa-2 + pLXSN and MIA-PaCa-2 + WT-GSK-3β cells.

Isoliquiritin is a natural product derived from licorice (Figure 10B). It is a flavonoid and has broad effects including anti-oxidant, anti-inflammatory, and anti-cancer properties [75]. It can activate TP53 in lung cancer cells [76]. Isoliquiritin has been shown to inhibit the in vitro invasiveness of PDAC cells [77]. The IC_50_ of isoliquiritin in MIA-PaCa-2 + pLXSN cells was approximately 600 nM, it was increased about 5-times in cells overexpressing WT-GSK-3β (Figure 10B, Table 1). In contrast, the gilteritinib IC_50_ in MIA-PaCa-2 + KD-GSK-3β cells was approximately 28 nM. 

Introduction of KD-GSK-3β to MIA-PaCa-2 cells resulted in decreases of IC_50_ values (increases in sensitivities) of the cells to all the tested chemotherapeutics. 

The highest increase of sensitivity was observed for isoliquiritin (over 21-fold) and doxorubicin (~6-fold).

In summary, comparing the effects of transfection of the cells with WT-GSK-3β to KD-GSK-3β, there was over 100-fold decrease of WT-GSK-3β-transfected MIA-PaCa-2 cells sensitivity to isoliquiritin, about 9-fold to doxorubicin, and much lower to other tested drugs.

### 3.8. Effects of WT-GSK-3β and KD-GSK-3β on Colony Formation in the Presence of Chemotherapeutic Drugs 

**Figure 11:**
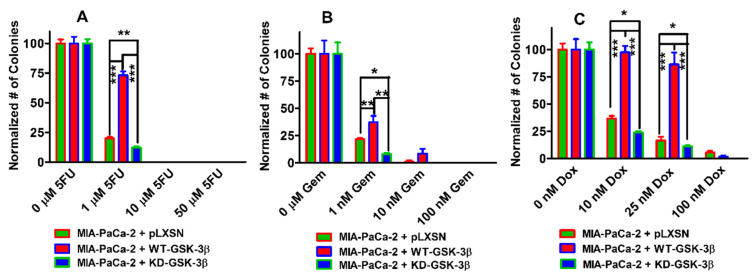
Effects of 5FU, gemcitabine and doxorubicin on colony formation of MIA-PaCa-2 + pLXSN, MIA-PaCa-2 + WT-GSK-3β, and MIA-PaCa-2 + KD-GSK-3β cells. The effects on colony formation in response to 5-FU (Panel (**A**)), gemcitabine (Gem), (Panel (**B**)) and doxorubicin (Dox) (Panel (**C**)) treatment on MIA-PaCa-2 + pLXSN (solid green bars), MIA-PaCa-2 + WT-GSK-3β (solid red bars), and MIA-PaCa-2 + KD-GSK-3β (solid blue bars). The MIA-PaCa-2 + WT-GSK-3β, MIA-PaCa-2 + KD-GSK-3β, and MIA-PaCa-2 + pLXSN cells in each panel were all examined at the same time period. In each condition, the cells were plated in 3 wells of a 6 well plate. The colony formation abilities were determined three times for each cell type and each treatment condition and similar results were observed. *** *p* < 0.0001, ** *p* < 0.005, and * *p* < 0.05.

To determine whether the changes in chemotherapeutic drug sensitivity observed by MTT analysis in MIA-PaCa2 + WT-GSK-3β, MIA-PaCa-2 + KD-GSK-3β, and MIA-PaCa-2 + pLXSN cells were also observed in larger scale cultures, the effects of three chemotherapeutic drugs were examined using colony formation analysis (Figure 11). We chose to examine the effects of 5FU, gemcitabine, and doxorubicin on colony formation as they are both used in therapy of PDAC patients, and doxorubicin is a commonly prescribed chemotherapeutic drug to treat various cancers including breast and leukemia patients. The data for each cell line and each drug treatment were normalized to the untreated control samples and compared. When MIA-PaCa-2 + WT-GSK-3β were plated in 5FU (Figure 11A), gemcitabine (Figure 11B), or doxorubicin (Figure 11C) more colonies were observed when MIA-PaCa-2 + pLXSN cells or MIA-PaCa-2 + WT-GSK-3β were plated under the same conditions than with MIA-PaCa-2 +KD-GSK-3β cells. Thus, WT-GSK-3β and KD-GSK-3β elicited positive and negative effects, respectively, on the sensitivity of MIA-PaCa-2 cells to chemotherapeutic drugs as determined by both MTT analysis and colony formation. 

### 3.9. Effects of WT-GSK-3β and KD-GSK-3β on Colony Formation in Presence of a GSK-3 inhibitor, the AMPK Activator Metformin, and the Nutraceutical Berberine

**Figure 12:**
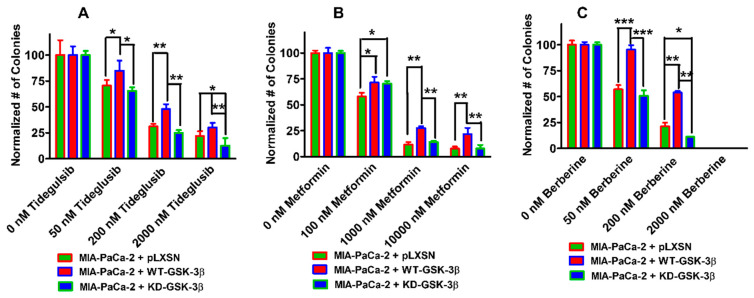
Effects of tideglusib, metformin and berberine on colony formation of MIA-PaCa-2 + pLXSN, MIA-PaCa-2 + WT-GSK-3β, and MIA-PaCa-2 + KD-GSK-3β cells. The effects on colony formation in response to tideglusib (Panel (**A**)), metformin (Panel (**B**)), and berberine (Panel (**C**)) treatment of MIA-PaCa-2 + pLXSN (solid green bars), MIA-PaCa-2 + WT-GSK-3β (solid red bars), and MIA-PaCa-2 + KD-GSK-3β (solid blue bars). The MIA-PaCa-2 + WT-GSK-3β, MIA-PaCa-2 + KD-GSK-3β, and MIA-PaCa-2 + pLXSN cells in each panel were all examined at the same time period. In each condition, the cells were plated in 3 wells of a 6 well plate. The colony formation abilities were determined three times for each cell type and each treatment condition and similar results were observed. *** *p* < 0.0001, ** *p* < 0.005, and * *p* < 0.05.

The abilities of MIA-PaCa-2 + WT-GSK-3β, MIA-PaCa-2 + KD-GSK-3β, and MIA-PaCa-2 + pLXSN to form colonies in the presences of a GSK-3 inhibitor, the type II diabetes drug metformin, and the nutraceutical berberine were also determined (Figure 12, Panels A, B, and C). We examined the effects of these three compounds on colony formation. Tideglusib is a GSK-3 inhibitor, which has been used in clinical studies; metformin is a common type-II diabetes drug; and berberine is a nutraceutical used in traditional medicine for various ailments. In general, more colonies were observed in MIA-PaCa-2 + WT-GSK-3β cells than either MIA-PaCa-2 + KD-GSK-3β or MIA-PaCa-2 + pLXSN cells, and less colonies were observed at higher drug concentrations. Thus, the MTT and colony formation assays yielded similar results indicating that expression of WT-GSK-3β enhanced resistance to a GSK-3 inhibitor, the type-II diabetes drug metformin, and the nutraceutical berberine. 

### 3.10. Effects of Introduction of WT-GSK-3β and KD-GSK-3β on Metabolic Activity in MIA-PaCa-2 Cells 

Cancer cells require a large amount of adenosine triphosphate (ATP) to grow rapidly. ATP is generated by glycolysis and mitochondrial oxidative phosphorylation. To determine the effects of GSK-3β on mitochondrial activity and metabolism, glycolysis and mitochondrial stress tests were performed on the various cells on the Seahorse instrument. The Seahorse instrument measures mitochondrial oxidative phosphorylation on the basis of the oxygen consumption rate (OCR), by performing real-time and live cell analysis. The instrument can also measure glycolysis by analyzing the extracellular acidification rate (ECAR). The effects of WT-GSK-3β, KD-GSK-3β on respiratory capacity were determined on MIA-PaCa-2 + pLXSN, MIA-PaCa-2 + WT-GSK-3β, and MIA-PaCa-2 + KD-GSK-3β cells. 

GSK-3 has been shown to be a mitochondria oxidative metabolism regulator in studies with B cells obtained from GSK-3 α and β knock-out mice [78], and Mv1Lu lung epithelial cells [79]. Studies have revealed that reduction of GSK-3 activity decreased cellular O_2_ consumption rate and it has been suggested that this may be a result of inhibition of respiratory complex IV activity in the absence of active GSK-3 [79]. 

**Figure 13:**
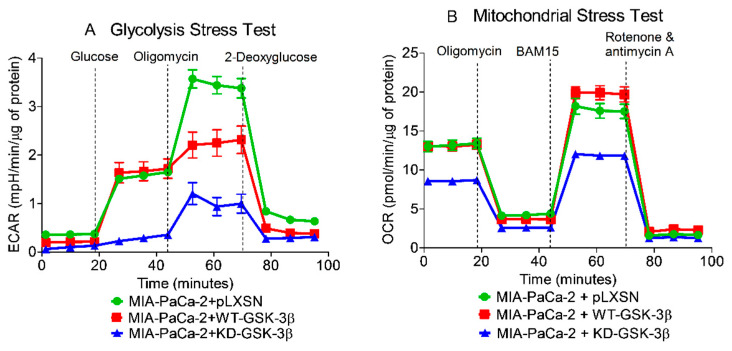
Effects of presence of WT-GSK-3β, KD-GSK-3β, and pLXSN on glycolysis and mitochondrial respiration.

**Figure 14:**
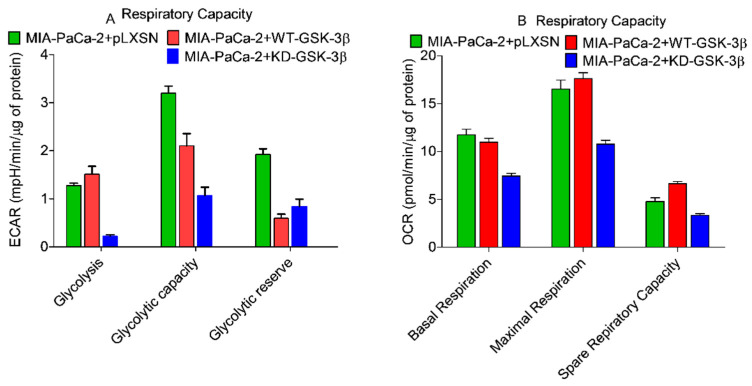
Effects of presence of WT-GSK-3β, KD-GSK-3β, and pLXSN on respiratory capacity.

On the other hand, it has been also shown that GSK-3 can down-regulate mitochondrial respiration by inhibition of pyruvate dehydrogenase and oxidative phosphorylation, by inhibiting respiratory chain complex I [80]. However, the effects of GSK-3β on PDAC mitochondrial activity are not well elucidated. The results presented here demonstrated that in MIA-PaCa-2 + pLXSN and MIA-PaCa-2 + WT-GSK-3β cells, all parameters of mitochondrial respiration were practically identical (Figure 13 and Figure 14) and differences between these cells were statistically insignificant. However, there was a significant difference between these cells and MIA-PaCa-2 + KD-GSK-3β cells. 

**Figure 15:**
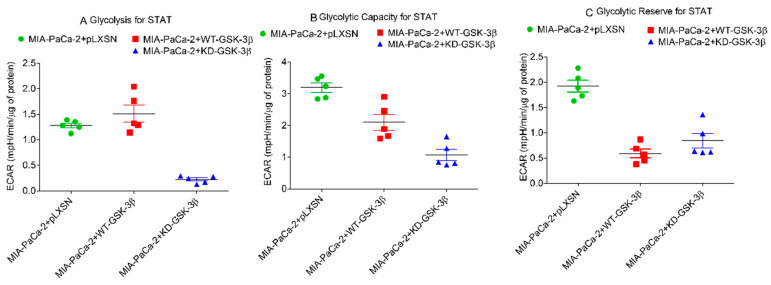
Effects of presence of WT-GSK-3β, KD-GSK-3β and pLXSN on glycolysis. Glycolysis for STAT, glycolytic capacity, and glycolytic reserve for STAT were measured by the Seahorse instrument. STAT is an abbreviation for statistics used in study which was the Mann–Whitney test.

The basal mitochondrial respiration was significantly lower in MIA-PaCa-2 + KD-GSK-3β cells than in MIA-PaCa-2 + WT-GSK-3β and MIA-PaCa-2 + pLXSN. Transfection of the cells with KD-GSK-3β reduced their maximal respiratory and respiratory capacity levels as compared to cells transfected with pLXSN or GSK-3β (MIA-PaCa-2 + pLXSN or MIA-PaCa-2 + WT-GSK-3β cells) (Figure 13 and Figure 14). Furthermore, MIA-PaCa-2 + KD-GSK-3β exhibited not only lower levels of mitochondrial oxidation but also many-fold reduced glycolytic activity compared to MIA-PaCa-2 + WT-GSK-3β or MIA-PaCa-2 + pLXSN cells (Figure 14 and Figure 15). The reduction of all glycolytic parameters (basal glycolysis, glycolytic capacity and the reserve) in MIA-PaCa-2 + KD-GSK-3β cells, presumably reflects the lower levels of glycolytic enzymes—a result of weaker stimulation of glycolysis by NF-κB which transcriptional activity is known to be regulated in GSK-3β-dependent manner (Figure 15).

In contrast to downregulation of GSK-3 (MIA-PaCa-2 + KD-GSK-3β cells), the overexpression of WT-GSK-3β had practically no effect on metabolic parameters of MIA-PaCa-2 cells except glycolytic capacity which was lower in these cells than in MIA-PaCa-2 + pLXSN cells. 

**Figure 16:**
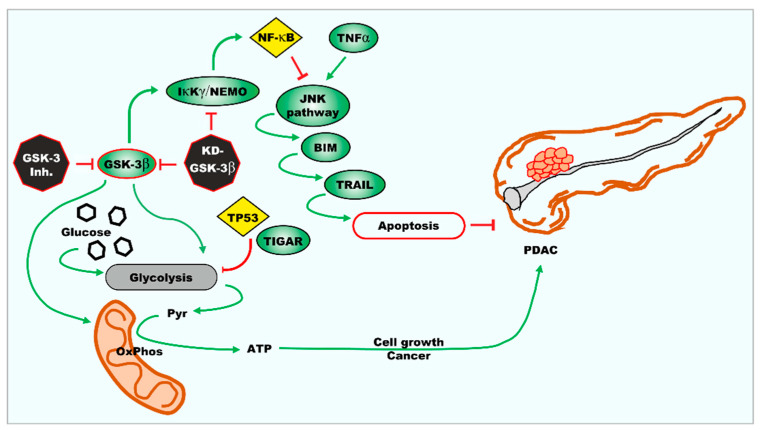
Interactions between the GSK-3β and glycolysis, metabolism, respiratory capacity, and drug sensitivity.

Inhibition of GSK-3 activity can decrease the metabolic properties of the cells reducing both glycolysis and mitochondrial respiration. An overview of the effects of GSK-3 on metabolic properties and the development of PDAC is presented in Figure 16.

### 3.11. Effects of Introduction of WT-GSK-3β, KD-GSK-3β, and pLXSN on Therapeutic Sensitivity of MCF-7 Breast Cancer Cells 

To ascertain whether GSK-3β may play different roles in various cancer types, we examined the effects of WT-GSK-3β, KD-GSK-3β, and pLXSN on the therapeutic sensitivity of MCF-7 breast cancer cells. Previously, we determined that introduction of KD-GSK-3β increased the resistance of MCF-7 cells to doxorubicin and tamoxifen, drugs which are used to treat ER+ breast cancers [37].

**Figure 17:**
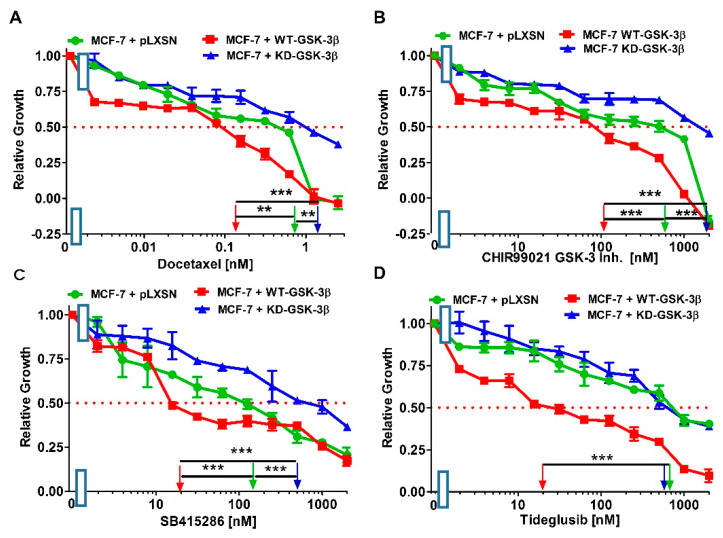
Docetaxel and GSK-3 inhibitors titrations on MCF-7 + pLXSN (green circles), MCF-7 + WT-GSK-3β (red squares), and MCF-7 + KD-GSK-3β (blue triangles) cells. (**A**) Docetaxel; (**B**) CHIR99021; (**C**) SB415286; and (**D**) Tideglusib. These experiments were all done on the same day and repeated 3 times and similar results were observed. *** *p* < 0.0001 and ** *p* < 0.005.

We examined the effects of WT-GSK-3β and pLXSN on the sensitivity of MCF-7 breast cancer cells to the chemotherapeutic drug docetaxel, and the GSK-3 inhibitors CHIR99021, SB415286, and tideglusib (Figure 17 and Table 2). Introduction of WT-GSK-3β into MCF-7 cells decreased the IC_50_ to docetaxel, CHIR99021, SB415286 and tideglusib 5.8-, 6-, 8-, and 44.4-fold, respectively, in comparison to MCF-7 + pLXSN cells.

We next examined the effects of KD-GSK-3β and pLXSN on the sensitivity of MCF-7 breast cancer cells. Introduction of KD-GSK-3β into MCF-7 cells increased the IC_50_ to docetaxel, CHIR99021, and SB415286, 1.9-, 3.1, and 3.1-fold, respectively (Figure 17, Table 2). In contrast, introduction of KD-GSK-3β decreased the IC_50_ to tideglusib 1.3-fold in MCF-7 + KD-GSK-3 in comparison to MCF-7 + pLXSN cells (Figure 17, Table 2). 

### 3.12. Effects of the Introduction of WT-GSK-3β, KD-GSK-3β, and pLXSN on Sensitivity of MCF-7 Breast Cancer Cells to the Type-II Diabetes Drug Metformin and the Nutraceutical Berberine

**Figure 18:**
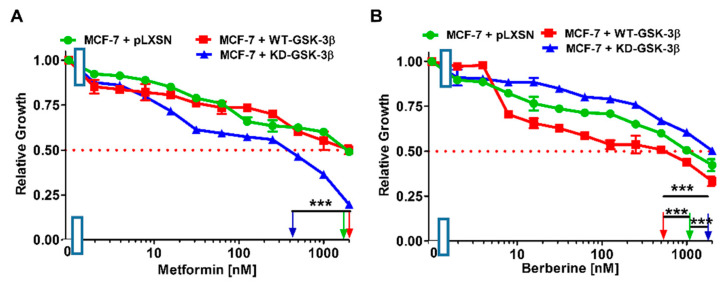
Metformin and Berberine titrations on MCF-7 + pLXSN (green circles), MCF-7 + WT-GSK-3β (red squares), and MCF-7 + KD-GSK-3β (blue triangles) cells. (**A**) Metformin; (**B**) Berberine. These experiments were all done on the same day and repeated 3 times and similar results were observed. *** *p* < 0.0001.

The effects of the type II diabetes drug metformin and the nutraceutical berberine were examined on the MCF-7 breast cancer cell line (Figure 18, Table 2). Suppression of GSK-3 has been observed to increase AMPK activity and autophagy in some cells [81]. Introduction of WT-GSK-3β into MCF-7 cells did not change the IC_50_ to metformin but it did increase the sensitivity to berberine 2.4-fold in comparison to MCF-7 + pLXSN cells (Table 2).

We next examined the effects of KD-GSK-3β and pLXSN, on the sensitivity of MCF-7 breast cancer cells to metformin and berberine. Introduction of KD-GSK-3β into MCF-7 cells decreased the IC_50_ to metformin 5-fold (Figure 18, Table 2). In contrast, introduction of KD-GSK-3β increased the IC_50_ to tideglusib 1.5-fold in comparison to MCF-7 + pLXSN cells (Figure 18, Table 2). 

### 3.13. Effects of the Introduction of WT-GSK-3β, KD-GSK-3β, and pLXSN on Sensitivity of MCF-7 Breast Cancer Cells to Tideglusib in Combination with Low Doses of Chemotherapeutic, Anti-Diabetes Drugs, and the Nutraceutical Berberine

**Figure 19:**
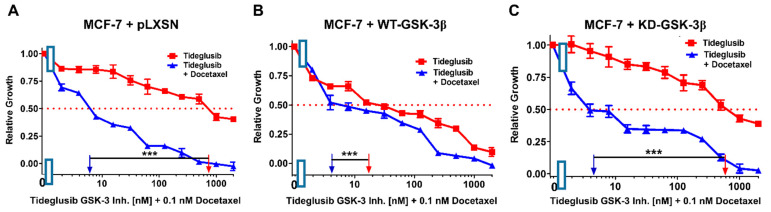
Effects of combining a constant concentration of 0.1 nM docetaxel on the tideglusib IC_50_. Tideglusib by itself (red squares) or tideglusib and a constant dose of 0.1 nM docetaxel (blue triangles on: (**A**) MCF-7 + pLXSN; (**B**) MCF-7 + WT-GSK-3β; and (**C**) MCF-7 + KD-GSK-3β cells). The experiments in (**A**–**C**) were all performed on the same day. They were repeated 3 times and similar results were observed. *** *p* < 0.0001.

**Figure 20:**
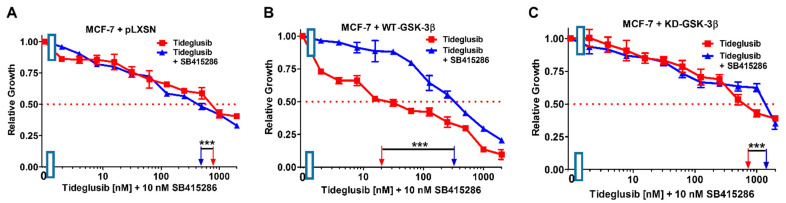
Effects of combining a constant concentration of 10 nM SB415286 on the tideglusib IC_50_. Tideglusib by itself (red squares) or tideglusib and a constant dose of 10 nM SB415286 (blue triangles on: (**A**) MCF-7 + pLXSN; (**B**) MCF-7 + WT-GSK-3β; and (**C**) MCF-7 + KD-GSK-3β cells). The experiments in (**A**–**C**) were all performed on the same day. They were repeated 3 times and similar results were observed. *** *p* < 0.0001.

**Figure 21:**
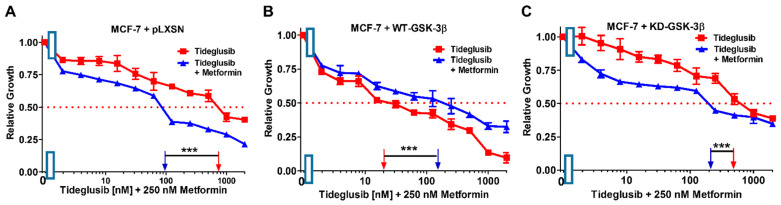
Effects of combining a constant concentration of 250 nM metformin on the tideglusib IC_50_. Tideglusib by itself (red squares) or tideglusib and a constant dose of 250 nM metformin (blue triangles on: (**A**) MCF-7 + pLXSN; (**B**) MCF-7 + WT-GSK-3β; and (**C**) MCF-7 + KD-GSK-3β cells). The experiments in (**A**–**C**) were all performed on the same day. They were repeated 3 times and similar results were observed. *** *p* < 0.0001.

**Figure 22:**
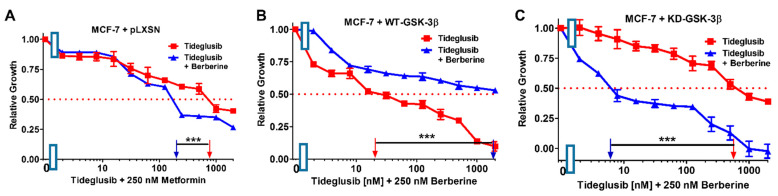
Effects of combining a constant concentration of 250 nM berberine on the tideglusib IC_50_. Tideglusib by itself (red squares) or tideglusib and a constant dose of 250 nM berberine (blue triangles on: (**A**) MCF-7 + pLXSN; (**B**) MCF-7 + WT-GSK-3β; and (**C**) MCF-7 + KD-GSK-3β cells). The experiments in (**A**–**C**) were all performed on the same day. They were repeated 3 times and similar results were observed. *** *p* < 0.0001.

As an alternative approach to examine the roles that GSK-3β may play in regulation of chemosensitivity, we investigated the effects of combination of the GSK-3 inhibitor tideglusib with low concentrations of docetaxel, SB415286, metformin, and berberine on MCF-7 + pLXSN, MCF-7 + WT-GSK-3β, and MCF-7 + KD-GSK-3β cells (Figure 19, Figure 20, Figure 21 and Figure 22 and Table 3). Addition of a low dose of docetaxel reduced the IC_50_ for tideglusib 77-, 4.5-, and 500-fold in MCF-7 + pLXSN, MCF-7 + WT-GSK-3β, and MCF-7 + KD-GSK-3β cells, respectively (Figure 18 and Table 3).

In contrast, the addition of a suboptimal concentration of the GSK-3 inhibitor SB415286 had more moderate effects on IC_50_ concentration of GSK-3 inhibitor tideglusib in both MCF-7 + pLXSN (1.6×↓) and MCF-7 + KD-GSK-3β (2.4×↑) cells but it did increase the tideglusib IC_50_ in MCF-7 + WT-GSK-3β cells 19.4-fold indicating that GSK-3 was playing a tumor suppressor role in these cells, and suppression of its activity increased therapeutic resistance (Figure 20, Table 3). 

The addition of a suboptimal concentration of metformin did reduce the IC_50_ concentration of the GSK-3 inhibitor tideglusib in both MCF-7 + pLXSN and MCF-7 + KD-GSK-3β cells, 5.1- and 10-fold, respectively, but increased the IC_50_ of tideglusib in MCF-7 + WT-GSK-3β cells 5.6-fold, indicating that GSK-3 was playing a tumor suppressor role in these cells, and suppression of its activity increased therapeutic resistance (Figure 21, Table 3).

Addition of a suboptimal concentration of berberine reduced the IC_50_ concentration of tideglusib in both MCF-7 + pLXSN and MCF-7 + KD-GSK-3β cells, 2.3- and 333-fold, respectively, but increased the IC_50_ in MCF-7 + WT-GSK-3β cells 111-fold indicating that GSK-3 was playing a tumor suppressor role in these cells, and berberine could not function to decrease cell growth in the presence of WT-GSK-3β expression (Figure 22, Table 3). Indeed, in MCF-7 + KD-GSK-3β cells, berberine was able to significantly inhibit cell growth at suboptimal concentrations. Thus, GSK-3 could play key roles in sensitivity of breast cancer cells to drugs, signal transduction inhibitors and nutraceuticals. 

## 4. Discussion

MIA-PaCa-2 cells have an activating mutation in the *KRAS* gene and a mutant *TP53* gene that encodes a gain of function (GOF) activity. Recently, regulatory loops have been observed in cells with mutant *TP53* and mutant *KRAS* genes, which result in elevated KRAS activity [47]. GSK-3β is a downstream signaling protein important in KRas-dependent growth and survival in mutant KRas-dependent cells such as MIA-PaCa-2 cells [47]. Thus, increased GSK-3β activity upon introduction of the WT-GSK-3β plasmid into MIA-PaCa-2 should make the cells more resistant to most drugs and signal transduction inhibitors. Suppression of GSK-3 activity with the KD-GSK-3β plasmid could decrease KRas-dependent proliferation.

Previously, we observed that introducing KD-GSK-3β into MCF-7 breast cancer cells increased their resistance to the chemotherapeutic drug doxorubicin and the hormonal based drug tamoxifen in comparison to MCF-7 cells that inherited WT-GSK-3β [37]. MCF-7 cells have WT *KRAS* and *TP53* and mutant *PIK3CA* genes. The presence of certain mutations in some cells may explain the ability of WT-GSK-3β to act like a tumor suppressor in some cells (e.g., MCF-7) but also act like a tumor promoter in other cells (e.g., MIA-PaCa-2). We have recently summarized the tumor promoter and tumor suppressor roles of GSK-3 [3,4]. 

In our previous studies with MCF-7 breast cancer cells, we compared the levels of GSK-3β protein and the extent of S9-phosphorylated GSK-3β protein, which is an indicator of its activity, by western blot analysis [37]. GSK-3β was dephosphorylated MCF-7 and MCF-7 + WT-GSK-3β cells upon treatment with doxorubicin indicating activation of GSK-3β. In contrast, GSK-3β was not activated in the MCF-7 +KD-GSK-3β cells upon doxorubicin treatment, and the cells were in fact more resistant to doxorubicin treatment [37]. 

In this study, we examined the effects of WT-GSK-3β and KD-GSK-3β on the sensitivity of MIA-PaCa-2 cells pancreatic cancer cells and MCF-7 breast cancer cells to a panel of chemotherapeutic drugs, signal transduction inhibitors, and nutraceuticals. Introducing WT-GSK-3β increased the IC_50_s of MIA-PaCa-2 pancreatic cancer cells to many drugs commonly used to treat PDAC, while WT-GSK-3 increased the sensitivity of MCF-7 breast cancer cells to certain drugs, signal transduction inhibitors, and nutraceuticals. MIA-PaCa-2 transfected with the pLXSN empty vector often displayed an intermediate sensitivity in comparison to cells transfected with either WT-GSK-3β or KD-GSK-3β. Thus, WT-GSK-3β was promoting resistance to these chemotherapeutic drugs and serving a tumor promoter role in MIA-PaCa-2 cells but a tumor suppressor in MCF-7 breast cancer cells. In contrast, KD-GSK-3β was promoting sensitivity (decreased the IC_50_s) to these drugs in MIA-PaCa-2 cells and serving a tumor suppressor role. KD-GSK-3β served a tumor promotor role (increased the IC_50_s) in MCF-7 cells.

Interestingly, introducing WT-GSK-3β increased the resistance to certain signal transduction inhibitors. MIA-PaCa-2 + WT-GSK-3β cells were more resistant to the EGFR/HER2, ALK/AXL/FLT3, KRAS, MEK1, GSK-3 inhibitors, and the mTORC1 blocker rapamycin, than MIA-PaCa-2 + KD-GSK-3β cells. 

Suppression of either mutant KRas or MEK1 also decreased the downstream effects of mutant KRas signaling. MIA-PaCa-2 cells with introduced WT-GSK-3β were more resistant to the PD0325901 MEK inhibitor than cells with the KD-GSK-3β or pLXSN. Thus, augmenting the level of WT-GSK-3β could increase the resistance of cells which contain mutant *KRAS* to MEK1 inhibitors. Treatment with MEK inhibitors have been shown to increase autophagy in pancreatic cancer with mutant *KRAS* genes. Suppression of autophagy was observed to synergize with MEK inhibitors [82]. Downstream of MEK is ERK. ERK can prime substrates for GSK-3β [83]. Some of substrates GSK-3β phosphorylates may alter the proliferation of the cells. Thus, WT-GSK-3 can alter the sensitivity to MEK inhibitors in PDAC cells with mutant *KRAS*. In contrast, we did not observe a significant difference in sensitivity to MEK inhibitors in MCF-7 breast cancer cells which have WT-*KRAS* upon treatment with MEK inhibitors by themselves [37]. However, treatment with MEK inhibitors did relieve the doxorubicin- and 4HT-resistance of the MCF-7 + KD-GSK-3β cells.

MIA-PaCa-2 cells with introduced WT-GSK-3β were also more sensitive to the BCL2/BCLXL inhibitor ABT-737 than cells transfected with KD-GSK-3β or pLXSN. Some of the targets of GSK-3 are BCL2-family members [84]. Thus, elevated expression of WT-GSK-3β made the cells more sensitive to the induction of apoptosis induced by ABT-737.

Similar observations have been observed in hematopoietic cells with a combination of small molecule inhibitors that target BCL2/BCLXL and PI3K/AKT signaling pathways [85]. Interactions between GSK-3 and the pro-apoptotic Bim molecule have been shown to increase the pro-apoptotic effects of BCL2/BCLXL inhibitors in human myeloid leukemia cells which were also treated with PI3K inhibitors.

Our results point to the effects that both WT-GSK-3β and KD-GSK-3β can have on sensitivity of certain pancreatic and breast cancer cells to chemotherapy and targeted therapy. These results are important as chemotherapeutic drugs can alter the activity of GSK-3β [3,86]. Therefore, without knowing the downstream consequences, suppression or activation of GSK-3β may change the sensitivity to targeted therapeutics. Clearly the role of GSK-3β in sensitivity to various drugs and signal transduction inhibitors should be further examined.

GSK-3 is an established therapeutic target and many compounds (e.g., lithium-chlo ride, tideglusib, and others) have been shown to suppress GSK-3 activity. Treatment with various GSK-3 inhibitors could influence the sensitivity to various drugs used to treat cancer patients. 

The inhibition of GSK-3 activity can also render PDAC cells more sensitive to chemotherapeutic drugs, signal transduction inhibitors, and nutraceuticals. In addition, we have previously observed that treatment of PDAC cells with low doses of metformin increases the sensitivity to multiple chemotherapeutic drugs and signal transduction inhibitors [87].

Predicting and determining which cancers will be sensitive to GSK-3 inhibition is very complicated. ER-negative breast cancers often have mutations in TP53 and other tumor suppressor and oncogenes such as *KRAS* which could influence GSK-3 beta expression and sensitivity to GSK-3 inhibitors. Often ER-negative breast cancers, especially triple-negative breast cancers (TNBC), are very drug resistant and may have EMT. GSK-3 plays critical roles in EMT due to interactions with the Wnt/β-catenin pathway. Inhibiting GSK-3 expression in certain TNBC decreased resistance to therapeutic drugs [88]. NF-κB is overexpressed and *KRAS* is mutant in certain TNBC (e.g., MDA-MB-231 cells), which may result in their sensitivity to GSK-3 inhibitors [89]. GSK-3 inhibitors have been proposed to regulate the cancer stem cell properties in TNBC [90]. Thus, further elucidation of the role of GSK-3 and its effective targeting may increase breast as well as pancreatic cancer therapy.

## Figures and Tables

**Table 1:** Effects of WT-GSK-3β, KD-GSK-3β, and pLXSN empty vector on sensitivity of MIA-PaCa-2 pancreatic cancer cells to chemotherapeutic drugs, signal transduction inhibitors, and nutraceuticals. The concentrations presented in the Table 1 represent the inhibitory concentration 50 (IC_50_) values (determined as previously described [37,49] for tested substances).

Drug/Agent↓	+pLXSN	+WT-GSK-3β	Fold Change WT vs. LXSN	+pLXSN	+KD-GSK-3β	Fold Change KD vs. LXSN	+WT- GSK-3β	+KD-GSK-3β	Fold Change WT vs. KD
5FU (nucleoside analogue)	6 µM	12 µM	2×↑	6 µM	3 µM	2×↓	12 µM	3 µM	4×↑
Gemcitabine (nucleoside analogue)	3.5 nM	5 nM	1.4×↑	3.5 nM	2 nM	1.8×↓	5 nM	2 nM	2.5×↑
Paclitaxel (microtubule binder)	1.2 nM	2.1 nM	1.8×↑	1.2 nM	1 nM	1.3×↓	2.1 nM	1 nM	2.1×↑
Docetaxel (microtubule binder)	0.15 nM	0.6 nM	4×↑	0.15 nM	0.08 nM	1.9×↓	0.6 nM	0.08 nM	7.5×↑
Cisplatin (DNA synthesis inh.)	110 µM	200 µM	1.8×↑	110 µM	65 µM	1.7×↓	200 µM	65 µM	3×↑
Oxaliplatin (DNA synthesis inh.)	0.4 µM	1.1 µM	2.8×↑	0.4 µM	0.03 µM	13.3×↓	1.1 µM	0.03 µM	36.7×↑
Irinotecan (topoisomerase inh.)	3 µM	4 µM	1.3×↑	3 µM	2 µM	1.5×↓	4 µM	2 µM	2×↑
Doxorubicin (topoisomerase inh.)	35 nM	55 nM	1.5×↑	35 nM	6 nM	5.8×↓	55 nM	6 nM	9.1×↑
Daunorubicin (topoisomerase inh.)	300 nM	800 nM	2.7×↑	300 nM	150 nM	2×↓	800 nM	150 nM	5.3×↑
Mitoxantrone (topoisomerase inh.)	18 nM	35 nM	1.9×↑	18 nM	6.5 nM	2.8×↓	35 nM	6.5 nM	5.4×↑
Etoposide (topoisomerase inh.)	800 nM	1 µM	1.3×↑	800 nM	550 nM	1.5×↓	1 µM	550 nM	1.8×↑
SB415286 (GSK-3α and β inh.)	120 nM	300 nM	2.5×↑	120 nM	15 nM	8×↓	300 nM	15 nM	20×↑
Tideglusib (GSK-3β inh.)	200 nM	5 µM	25×↑	200 nM	70 nM	2.9×↓	5 µM	70 nM	71.4×↑
BIO (GSK-3α and β inh.)	300 nM	800 nM	2.7×↑	300 nM	150 nM	2×↓	800 nM	150 nM	5.3×↑
CHIR99021 (GSK-3α and β inh.)	350 nM	650 nM	1.9×↑	350 nM	170 nM	2.1×↓	650 nM	170 nM	3.8×↑
ARRY-543 (EGFR/HER2 inh.)	400 nM	2 µM	5×↑	400 nM	40 nM	10×↓	2 µM	40 nM	50×↑
ARS-1620 (KRas inh.)	5 nM	30 nM	6×↑	5 nM	3 nM	1.7×↓	30 nM	3 nM	10×↑
PD0325901 (MEK1 inh.)	125 nM	280 nM	2.3×↑	125 nM	35 nM	3.6×↓	280 nM	35 nM	8×↑
Rapamycin (mTORC1 blocker)	0.6 nM	2 nM	3.3×↑	0.6 nM	0.28 nM	2.1×↓	2 nM	0.28 nM	7.1×↑
ABT-737 (BCL2/BCLXL inh.)	350 nM	7 nM	50×↓	350 nM	350 nM	1 ×	7 nM	350 nM	50×↓
Metformin (Activation of AMPK)	7 µM	9 µM	1.3×↑	7 µM	6 µM	1.2×↓	9µM	6 µM	1.7×↓
Berberine (multiple targets, mTORC1, NF-κB, AMPK)	1.2 µM	2 µM	1.7×↑	1.2 µM	600 nM	2×↓	2 µM	600 nM	3.3×↑
Chloroquine (autophagy inh. apoptosis inducer)	0.9 µM	20 µM	22×↑	0.9 µM	0.9 µM	1×	20 µM	0.9 µM	22×↑
OTX008 (Galectin-1 inh.)	1.1 µM	1.3 µM	1.2×↑	1.1 µM	19 nM	57.9×↓	1.3 µM	19 nM	68×↑
Tiplaxtinin (Serpine-1 inh.)	20 nM	200 nM	10×↑	20 nM	20 nM	1×	200 nM	10 nM	20×↑
Vismodegib (Hh inh.)	5 µM	8 µM	1.8×↑	5 µM	5 µM	1×	8 µM	5 µM	1.8×↑
Gilteritinib (AXL, ALK, FLT3 inh.)	550 nM	700 nM	1.3×↑	550 nM	400 nM	1.4×↓	700 nM	400 nM	1.8×↑
Isoliquiritin (multiple targets)	600 nM	3 µM	5×↑	600 nM	28 nM	21.4×↓	3 µM	28 nM	107×↑

**Table 2:** Effects of introduction of pLXSN, WT-GSK-3β, and KD-GSK-3β on therapeutic sensitivity of MCF-7 breast cancer cells determined by MTT analysis (as described previous and [49]).

Drug, Signal Transduction Inhibitor or Nutraceutical↓	+pLXSN	+WT-GSK-3β	Fold Difference Compared to pLXSN	+KD-GSK-3β	Fold Difference Compared to pLXSN
Docetaxel	0.7 nM	0.12 nM	5.8×↓	1.3 nM	1.9×↑
CHIR99021	600 nM	100 nM	6×↓	1900 nM	3.1×↑
SB415286	160 nM	20 nM	8×↓	500 nM	3.1×↑
Tideglusib	800 nM	18 nM	44.4×↓	600 nM	1.3×↓
Metformin	2000 nM	2000 nM	1 ×	400 nM	5×↓
Berberine	1200 nM	550 nM	2.4×↓	1800 nM	1.5×↑

**Table 3:** Effects of pLXSN, WT-GSK-3β, and KD-GSK-3β on sensitivity of MCF-7 breast cancer cells to treatment with the GSK-3 inhibitor tideglusib in combination with drugs, signal transduction inhibitors, and a nutraceutical as determined by MTT analysis (as described previous and [49]).

Tideglusib in Combination with Drugs, Signal Transduction Inhibitor or Nutraceutical↓	+pLXSN	Fold Difference Compared to Tideglusib Alone	+WT-GSK-3β	Fold Difference Compared to Tideglusib Alone	+KD-GSK-3β	Fold Difference Compared to Tideglusib Alone
None	800 nM	-	18 nM	-	600 nM	-
0.1 nM Docetaxel	6 nM	77×↓	4 nM	4.5×↓	4 nM	150×↓
250 nM SB415286	500 nM	1.6×↓	350 nM	19.4×↑	1300 nM	2.2×↑
Metformin	100 nM	8×↓	100 nM	5.6×↑	200 nM	3×↓
Berberine	200 nM	4×↓	2000 nM	111×↑	6 nM	100×↓
